# Commentary: Sensation seeking and adaptation in parabonauts

**DOI:** 10.3389/fpsyg.2024.1482261

**Published:** 2025-01-06

**Authors:** Alan Silburn

**Affiliations:** School of Medicine, Western Sydney University, Campbelltown, NSW, Australia

**Keywords:** extreme environment, personality profiling, recruitment, adaptation, big five personality traits, human performance

## Discussion

A human's physical, social, and psychological attributes are all challenged when functioning in extreme and unusual environments.

Thus, individuals inhabiting these environments must possess certain characteristics to ensure performance and successful adaptation to their situations and surroundings (Barnett and Kring, 2003; Bishop, 2004). The following will define the factors contributing to an extreme environment and how they affect the humans inhabiting it. With this definition established, an assessment will be conducted to determine an individual's suitability for inhabiting diverse environments and how mitigation strategies at recruitment may enhance human functioning when inhabiting extreme environments.

An extreme environment can be defined as an environment that poses an increased risk to life or is hazardous to a subjected organism to which it is not suited (Boyd et al., 2016). For example, sea life that has evolved to thrive in the hyperbaric conditions of the ocean's abyss, an environment humans consider extreme, is optimally suited to sustain life, and leaving this environment would prove fatal (Wingfield et al., 2011). However, unlike others, the human species has become proficient at tailoring the conditions to suit our natural physiological demands to be able to thrive in almost every extreme environment accessible to us. These conditions, or physical characteristics, deviate from the requirements to maintain human functioning at an optimal level. These extreme conditions are often associated with high or low temperatures, isolation, alterations in light-dark cycling and circadian rhythms, physical discomfort, reduced visibility, or stresses of sudden disaster (Suedfeld, 2001). Although the human species has occupied and thrived in extreme environments (Barnett and Kring, 2003), these harsh and demanding conditions significantly impact the physical and psychological functioning of humans, which not all humans may be suited to endure.

Extreme environments come in various forms, extending beyond naturally harsh physical landscapes to include situations shaped by social, cultural, and even organizational pressures. For instance, while the cold, isolation, and oxygen-deprived conditions of polar regions or high-altitude mountains are quintessential examples, other environments, such as those affected by climate change-induced heatwaves, rising sea levels, and sudden natural disasters, also qualify as extreme due to the immediate threat they pose to human health and safety (Thompson et al., 2023). Climate change is increasingly creating environments that test human resilience and adaptability by directly exposing individuals to hazardous conditions and disrupting entire ecosystems and communities (Myers and Patz, 2009). In this sense, environments that were once manageable for human habitation may shift into the extreme category due to evolving climate pressures.

Similarly, social and cultural dynamics can produce extreme environments that challenge human functioning in less visible but equally impactful ways. For example, individuals operating in active war zones face profound psychological and emotional stress in addition to physical dangers. In these settings, people must cope with constant threats of violence, potential loss of life, and the trauma associated with witnessing or experiencing violence firsthand. The unpredictable nature of war, combined with the intensity of emotional strain and limited access to necessities, creates an environment that severely tests psychological resilience. Just as physically harsh landscapes challenge endurance, warzones challenge mental fortitude as individuals navigate a high-stakes environment where their safety, emotional stability, and wellbeing are persistently under threat (Fontana and Rosenheck, 1999).

Given the diversity of extreme environments, certain personality traits may enhance an individual's ability to function and adapt successfully within these varied settings. Traits such as high stress tolerance, emotional stability, and adaptability are particularly valuable for individuals facing physically harsh conditions, such as those found in space or disaster zones (Bartone et al., 2018). In socially adverse environments, such as conflict-ridden regions, qualities such as resilience, strong interpersonal skills, and a high capacity for empathy are crucial (Fletcher and Sarkar, 2013). While physical endurance may be critical in one setting, psychological resilience and adaptability may be more important in another, indicating that specific environments demand unique personality profiles to ensure optimal performance and wellbeing. These attributes are further presented in Table 1.

**Table 1:** Personality profiles in extreme environments.

**Environment category**	**Examples**	**Suitable personality traits**	**Potential impacts on health and performance**	**References**
Physically harsh	High altitude, deep sea, space, polar regions	Resilience, adaptability, high-stress tolerance, physical and mental endurance, patience, precision, problem-solving, self-sufficiency, high discomfort tolerance	**Positive:** Enhanced coping, improved decision-making under pressure, greater resilience. **Negative:** Altitude sickness, hypoxia, decompression sickness, isolation stress, muscle atrophy, radiation exposure, frostbite, sleep disruptions; impacts concentration and physical stamina.	Le Roy et al., 2023; Leach, 2016; Sarris, 2006
Socially adverse	Combat zones, disaster response, high-risk civilian roles (e.g., firefighters, paramedics)	Strong emotional resilience, high-stress tolerance, empathy, quick decision-making, adaptability, courage, teamwork, controlled risk-taking	**Positive:** Ability to build trust, navigate complex social dynamics, and maintain morale. **Negative:** PTSD, trauma, physical exhaustion, emotional strain, fatigue, physical injury risk	Smith and Barrett, 2018
Culturally distinct	Remote or isolated communities, field research in harsh regions	Adaptability, community orientation, strong endurance, cultural sensitivity, patience, empathy, openness, and self-motivation.	**Positive:** Successful integration into the community, enhanced understanding of the environment and its challenges. **Negative:** Strain from harsh climates, isolation stress, and culture shock	Thomas and Clark, 2007
Isolated/confined	Polar stations, spaceflight, submarines, hyperbaric research	Low extraversion, emotional stability, adaptability, resilience	**Positive:** Ability to cope with limited social interaction, focus on tasks, and adapt to unusual living conditions. **Negative:** Risk of social withdrawal, psychological stress due to confinement, difficulty readjusting to social environments upon return.	Van Wijk, 2022
High-stakes/high-risk	Military, deep-sea diving, extreme sports	Conscientiousness, emotional stability, low neuroticism, high stress tolerance, risk tolerance, perseverance, high physical resilience	**Positive:** Enhanced performance under pressure, adherence to safety protocols, and effective decision-making in critical situations. **Negative:** Potential for psychological trauma, burnout, physical injury if safety measures are not followed, injury risk, and exhaustion.	Devonport et al., 2022; Fontana and Rosenheck, 1999

For many of us, having access to modern conveniences that provide a comfortable and stable environment in our homes and places of work bestows an unsurpassable quality of life (Davis and Buskist, 2008, p. 210–218). However, some select individuals choose to live and operate in the less-than-favorable conditions of extreme environments. Individuals who choose to live and work in extreme environments are often driven by a sense of purpose, adventure, or cultural tradition. Professions like military service, deep-sea commercial diving, and scientific field research attract those committed to impactful work in high-stakes settings, while adventurers, such as mountaineering guides and polar researchers, are drawn to pushing personal limits and exploring the unknown. Cultural traditions also play a role; Indigenous groups like the Inuit in the Arctic, Bedouins in the desert, and Sherpa of Nepal have adapted to their harsh landscapes for generations, finding identity and purpose in these environments. These individuals must contain certain characteristics that make them uniquely suited to adapt to the complex, challenging, and often dangerous activities that are required in extreme environments (Davis and Buskist, 2008, p. 210–218). Therefore, during the recruitment process, a personality profile should be formulated to suggest if a person is suitable for the required environmental deployment.

Personality profiling in personnel recruitment has been utilized by countless organizations to determine if that person has “the right stuff” by weighing them against predetermined criteria, allowing a select-out/in approach to personnel selection. To gather this information, the selected cohort is asked to complete a personality questionnaire based on the International Personality Item Pool that addressed their “Big 5′ personality dimensions, as these traits are the most commonly used to model personality in academic psychology (Goldberg et al., 2006).” These dimensions of personality determine a person's degree of neuroticism, extraversion, agreeableness, conscientiousness, and openness to experience (Goldberg et al., 2006).

Following the questionnaire, individual results are displayed and classified relative to the standard deviation of the cohort for the five categories of personality dimensions. Scores were determined as low, moderately low, average, moderately high, and high, depending on the deviation from the mean.

After the data is collated, the select-out phase excluded those with the risk of poor psychosocial adaptation and inconsistencies in task performance. Commonly, those excluded meet psychiatric criteria or may be at high risk for such disorders, present with inadequate preparation, or possess a problematic life history, as antisocial or unpredictable behaviors may jeopardize the intended objective (John Paul, 2014). Regarding the questionnaire results, those who scored low on agreeableness are deemed as being highly critical and aggressive, and those low on conscientiousness are deemed as being impulsive and disorganized, thus posing a risk of antisocial or unpredictable behaviors, and are subsequently excluded from the recruitment process.

Secondly, criteria are utilized to select-in individuals with desirable characteristics in an attempt to predict the adaptation, reliability, and performance in these environments (John Paul, 2014). These desirable attributes are noticeable in people who are conscientious, emotionally stable, less anxious, and who tolerate stress better than the general population (Collado et al., 2018) and thus would therefore not be as susceptible to depression, irritability, aggressive behavior, insomnia, difficulty in concentration, or absentmindedness common to those in extreme or isolated environments (John Paul, 2014). Hence, the select-in criteria are often set to those as having high levels of conscientiousness, high levels of openness to experience, and low levels of neuroticism, as these individuals would reflect the desirable attributes of being careful and diligent, emotionally stable, and would be more flexible in dealing with non-traditional challenges and problem-solving (Parr et al., 2016).

The final personality dimension of extroversion was considered a neutral classification that could be used in selection to reflect the nature of the mission and the type of extreme environment in question. For example, those with high extroversion results are suited for group work such as expedition teams, multi-agency deployments, or leadership roles. In contrast, this need for interpersonal and organizational connection is a flaw in environments of low social contact, such as polar stations, spaceflight, hyperbaric research, and other individual or small team missions that are more suited to those of a low extroversion level.

## Conclusion

By their nature, extreme environments place extraordinary demands on human capabilities across physical, social, and psychological domains.

Therefore, identifying individuals with the “traits” is essential for mission success and individual wellbeing. As part of a comprehensive selection process, personality profiling plays a vital role in assessing an individual's suitability for these challenging contexts. By evaluating traits such as agreeableness, conscientiousness, emotional stability, and adaptability, organizations can better predict an individual's ability to not only survive but also thrive in extreme environments.
